# Limitations of dual‐fluorescent HIV reporter viruses in a model of pre‐activation latency

**DOI:** 10.1002/jia2.25425

**Published:** 2019-12-19

**Authors:** Youry Kim, Paul U Cameron, Sharon R Lewin, Jenny L Anderson

**Affiliations:** ^1^ Department of Microbiology and Immunology The University of Melbourne at the Peter Doherty Institute for Infection and Immunity Melbourne Victoria Australia; ^2^ The Peter Doherty Institute for Infection and Immunity The University of Melbourne and Royal Melbourne Hospital Melbourne Victoria Australia; ^3^ Department of Infectious Diseases Alfred Hospital and Monash University Melbourne Victoria Australia

**Keywords:** HIV, dual‐fluorescent reporter, latency, pre‐activation model of HIV, latency establishment, HIV cure, immunology, virology

## Abstract

**Introduction:**

HIV latency can be established *in vitro* following direct infection of a resting CD4+ T cell (pre‐activation latency) or infection of an activated CD4+ T cell which then returns to a resting state (post‐activation latency). We modified a previously published dual‐fluorescent reporter virus seeking to track the establishment and reactivation of pre‐activation latency in primary CD4+ T cells.

**Methods:**

A previously published dual‐fluorescent reporter virus was modified so that expression of enhanced green fluorescent protein (GFP) was under control of the elongation factor 1 alpha (EF1α) promoter to detect latent infection, and E2 crimson (E2CRM) was under control of the *nef* promoter to detect productive infection. NL4.3 that expressed GFP in place of *nef* was used as a positive control. We infected the Jurkat T‐cell line and primary CD4+ T cells that were either unstimulated or stimulated with either the chemokine CCL19 or phytohaemagglutinin (PHA)/IL‐2 and quantified the expression of both fluorescent proteins by flow cytometry. The study was carried out over a period of two years from September 2016 to October 2018.

**Results and Discussion:**

Expression of both fluorophores was detected following infection of the Jurkat T‐cell line while only low levels of the latent reporter were observed following infection of primary CD4+ T cells. In unstimulated and CCL19‐treated CD4+ T cells, expression of the GFP latent reporter, increased after further activation of the cells with PHA/phorbol 12‐myristate 13‐acetate (PMA).

**Conclusions:**

Our findings demonstrate that the EF1α promoter has poor constitutive expression in resting CD4+ T cells. Therefore, dual‐fluorescent reporter viruses with the EF1α promoter may underestimate the frequency of latent infection in resting CD4+ T cells.

## 1 Introduction

The main barrier to finding a cure for HIV infection is the persistence of latently infected cells in HIV‐infected individuals on antiretroviral therapy (ART) 1, 2, 3. *In vitro*, latent infection can be established through multiple pathways: direct infection of resting CD4+ T cells (pre‐activation latency 4), infection of an activated cell which survives and reverts to a resting state (post‐activation latency) 5 or infection of a cell transitioning from an effector memory to central memory phenotype 6, 7. Given the low frequency of latently infected cells *in vivo* 8 insights into latency are often obtained through the use of *in vitro* models of latent infection established in primary T cells or T‐cell lines (reviewed in 9).

In recent years, dual‐fluorescent reporter viruses have emerged as a common tool to study latency *in vitro* 10, 11, 12 by including two fluorescent reporters – a latent reporter that is under the control of an HIV LTR‐independent promoter that is constitutively expressed following integration and a productive reporter that is expressed following transcription from the HIV LTR. Duo‐Fluo is a previously reported virus that contains mCherry under the control of the elongation factor 1α (EF1α) promoter as the latent reporter and enhanced green fluorescent protein (GFP) as the reporter for productive infection, both inserted into the *nef* gene 11. One potential limitation of the Duo‐Fluo virus is the possibility of recombination of the two reporters during reverse transcription due to the matching amino and carboxyl termini 13. This limitation was recently addressed in a new virus HIV_GKO_, whereby mCherry was replaced with Kusabira orange (mKO2) and GFP was switched to a codon‐switched GFP protein (csGFP) 14.

In order to enhance infectivity in primary cells and to enhance the detection of latent virus established by direct infection (pre‐activation latency), we designed a new dual‐fluorescent reporter virus. We modified Duo‐Fluo and restored *env* and *vpr* to wild type sequences and changed the reporter proteins to GFP (latent) and E2 crimson (productive infection), naming it DuoAdvance. Following direct infection of resting cells stimulated with CCL19 4, 15, we found low constitutive expression of GFP (the latent reporter), despite successfully establishing latent infection. We conclude that dual‐fluorescent reporter viruses may underestimate the frequency of latently infected cells in resting CD4+ T cells.

## 2 Methods

### 2.1 Cell line culture

The Jurkat T‐cell line was cultured in RPMI‐1640 (RF10) and human embryonic kidney (HEK) 293T cells and TZM‐bl cells (cell lines: NIH AIDS Reference Reagent Program) were cultured in Dulbecco's modified eagle medium (DMEM10) at 37°C, 5% CO_2_. All media was supplemented with 10% FBS, 100 U/mL penicillin, 100 μg/mL streptomycin and 29.2 mg/mL L‐glutamine.

### 2.2 Preparation of E2 crimson fluorescent protein plasmids, NL4.3‐EGFP

E2 crimson fluorescent plasmid, NL4.3‐EGFP and Duo‐Fluo plasmids were transformed in Stbl‐2 cells and plated on Luria broth (100 μg/mL ampicillin) or terrific broth (TB) (100 μg/mL kanamycin for Duo‐Fluo). Single colonies were cultured for 16 hours and plasmids prepared using QIAprep Spin Miniprep Kits (QIAGEN, Hilden, Germany) as per the manufacturer's instructions.

### 2.3 DuoAdvance plasmid generation

A 6668bp DNA plasmid insert containing the modified DuoAdvance sequence was synthesized by GenScript (GenScript, Piscataway, NJ). The DuoAdvance DNA plasmid insert was ligated into the Duo‐Fluo plasmid using T4 DNA ligase. Plasmids were prepared using a Maxi Kit (QIAGEN, Hilden, Germany) and ligation confirmed using combined *NdeI* and *PacI* digestion for 2.5 hours at 37°C followed by 1 hour at 50°C with *BssHII,* resolved on a 1.2% gel.

### 2.4 Preparation of virus stocks and determining infectivity

Viral stocks of DuoAdvance, Duo‐Fluo and NL4.3‐EGFP were prepared from transfection of HEK293T cells with each respective plasmid, concentrated and used in all experiments as previously described 15.

The infectivity of viral stocks was performed using a TZM‐bl assay. TZM‐bl cells were plated and infected with 10‐fold dilutions of viral stocks in triplicate. Negative and positive control wells using media and a reference virus with known infectivity were also included respectively. 50% tissue culture infectious dose (TCID_50_) was measured using luciferase readout and calculated using the Reed‐Muench method 16.

### 2.5 Infection of Jurkat T‐cell line

Jurkat T cells were infected with concentrated DuoAdvance virus, Duo‐Fluo virus, NL4.3‐EGFP virus at a TCID_50_ of 0.5 in an infection volume of 100 μL. The integrase inhibitor Raltegravir (1 μmol/L) or non‐nucleoside reverse transcriptase inhibitor Nevirapine (10 μmol/L) (Selleck Chemicals, Houston, TX) were added to cells 30 minutes before infection and then maintained in cultures throughout the remaining experiment. Cells were infected for 2 hours, washed, cultured for three days and then fixed in 1% formaldehyde for flow cytometry. Samples were analysed on a BD LSR Fortessa using FACSDIVA software (BD Biosciences, San Jose, CA). Data were analysed using FlowJo v10 (FlowJo LLC, Ashland, OR).

### 2.6 Isolation and infection of resting CD4+ T cells

Peripheral blood mononuclear cells (PBMCs) and resting CD4+ T cells were isolated from whole blood from healthy donors as described previously 4 and cultured in three different conditions; unactivated, 100 nmol/L recombinant human CCL19/MIP‐3β (In Vitro Technologies, Vic, AUS) treated and PHA/IL‐2 activated. Untreated and treated primary CD4+ T cells were typically infected with 100 µL of DuoAdvance or NL4.3‐EGFP (both at TCID_50_ 0.5‐1) per 1 × 10^6^ cells for 2 hours. Cells were also spinoculated (1200×g, 90 minutes, room temperature) followed by a further 30 minutes incubation. Cells were then washed and resuspended in RF10 plus 1 U/mL IL‐2 and cultured for six days.

For reactivation studies, the E2CRM− fraction of cells were sorted out from CCL19‐treated CD4+ T cells infected with DuoAdvance using a BD FACSAria Fusion (BD, Franklin Lakes, NJ) and then reactivated by the addition of 10 μg/mL PHA+10 nmol/L PMA, or DMSO diluent negative control, for three days in the presence of 1 μmol/L raltegravir. After reactivation, cells were fixed in 1% formaldehyde and run on a BD LSR Fortessa using bandpass filters for E2CRM (670/30 Yellow) and GFP (530/30 Blue) plus FACSDIVA software. Results were analysed using FlowJo v10.

### 2.7 Data analysis

FlowJo results were graphed using GraphPad Prism (version 7, GraphPad Software, La Jolla, CA). Statistical testing was not appropriate as the small sample size (n < 6) precluded the use of non‐parametric tests. Due to the small sample size, testing normality cannot be performed, thus parametric t‐tests were also not used. Additionally, as the percentage fluorescence data points appeared skewed, this further suggests that a parametric t‐test was not appropriate. Therefore, we have only reported the mean, range and fold‐difference between means.

## 3 Results and discussion

### 3.1 DuoAdvance HIV reporter virus can establish latent and productive infection in a T‐cell line

**Figure 1:**
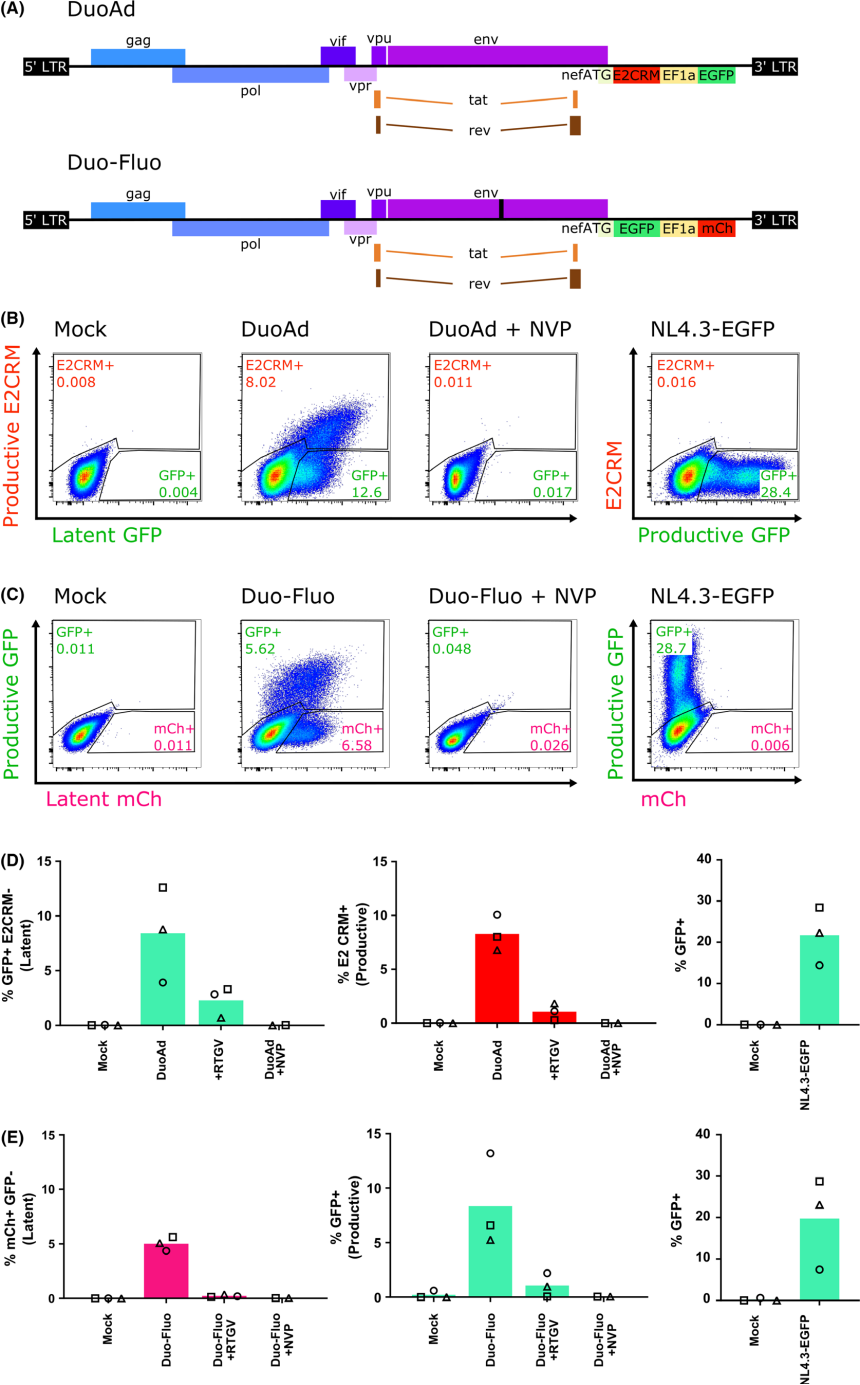
DuoAdvance infection of the Jurkat T‐cell line generates latent and productively infected cells similar to Duo‐Fluo. (**A**) Schematic of the DuoAdvance (DuoAd) dual‐fluorescent reporter construct virus (top) and Duo‐Fluo dual‐fluorescent reporter construct (bottom). DuoAd contains an EF1α promoter driven enhanced green fluorescent protein (EGFP) marker for latent cells and an E2 Crimson (E2CRM) marker under the control of the HIV LTR that serves as the productive marker. This construct also contains wildtype HIV *env* and *vpr* sequence. Jurkat T cells were infected with either DuoAd (**B, D**) or Duo‐Fluo (**C, E**) for three days in the presence or absence of nevirapine (NVP) or raltegravir (RTGV) and expression of EGFP (GFP), E2CRM or mCherry (mCh) quantified by flow cytometry. NL4.3‐EGFP virus was used as a positive control for productive infection and expression of GFP (**B‐E**). Representative flow plots and gates are shown in b and c. In D‐F, each symbol represents an independent experiment and columns represent the mean.

We first compared infection of the Jurkat T‐cell line using DuoAdvance, the original Duo‐Fluo (Figure 1A) and NL4.3‐GFP as a comparison for GFP productive infection reporter expression. Cells were infected at 0.5 TCID_50_ per cell as titration studies found comparable levels of latent infection of CCL19‐treated CD4+ T cells using DuoAdvance at 0.5‐2 TCID_50_ after 72 hours (data not shown)**.** Following infection of the Jurkat T‐cell line with DuoAdvance, latently infected (GFP+E2CRM−) and productively infected cells (E2CRM+) were detected (Figure 1). All E2CRM+ cells were considered productively infected irrespective of GFP expression. The mean fluorescence intensity of GFP as a latency reporter following DuoAdvance infection (mean (range) = 8.43 (3.92 to 12.60)%) was below the intensity observed after infection with NL4.3‐EGFP where GFP is a productive reporter (mean (range) = 21.70 (14.40 to 28.40)%) (Figure 1B,D). Infection with DuoAdvance and Duo‐Fluo (Figure 1A) resulted in a mean frequency of 8.43% (range = 3.93 to 12.60%) and 5.02% (range = 4.36 to 5.62%) latently infected cells respectively (Figure 1B‐E). The number of productively infected cells was also comparable between both viruses (Figure D,E). Nevirapine or raltegravir successfully inhibited infection of Jurkat T cells by both viruses (Figure 1B‐E). As DuoAdvance and Duo‐Fluo were comparable in their infection of the Jurkat T‐cell line, DuoAdvance therefore showed promise as a new dual‐fluorescent reporter virus.

### 3.2 Minimal constitutive expression of EF1α following infection of resting cells

**Figure 2:**
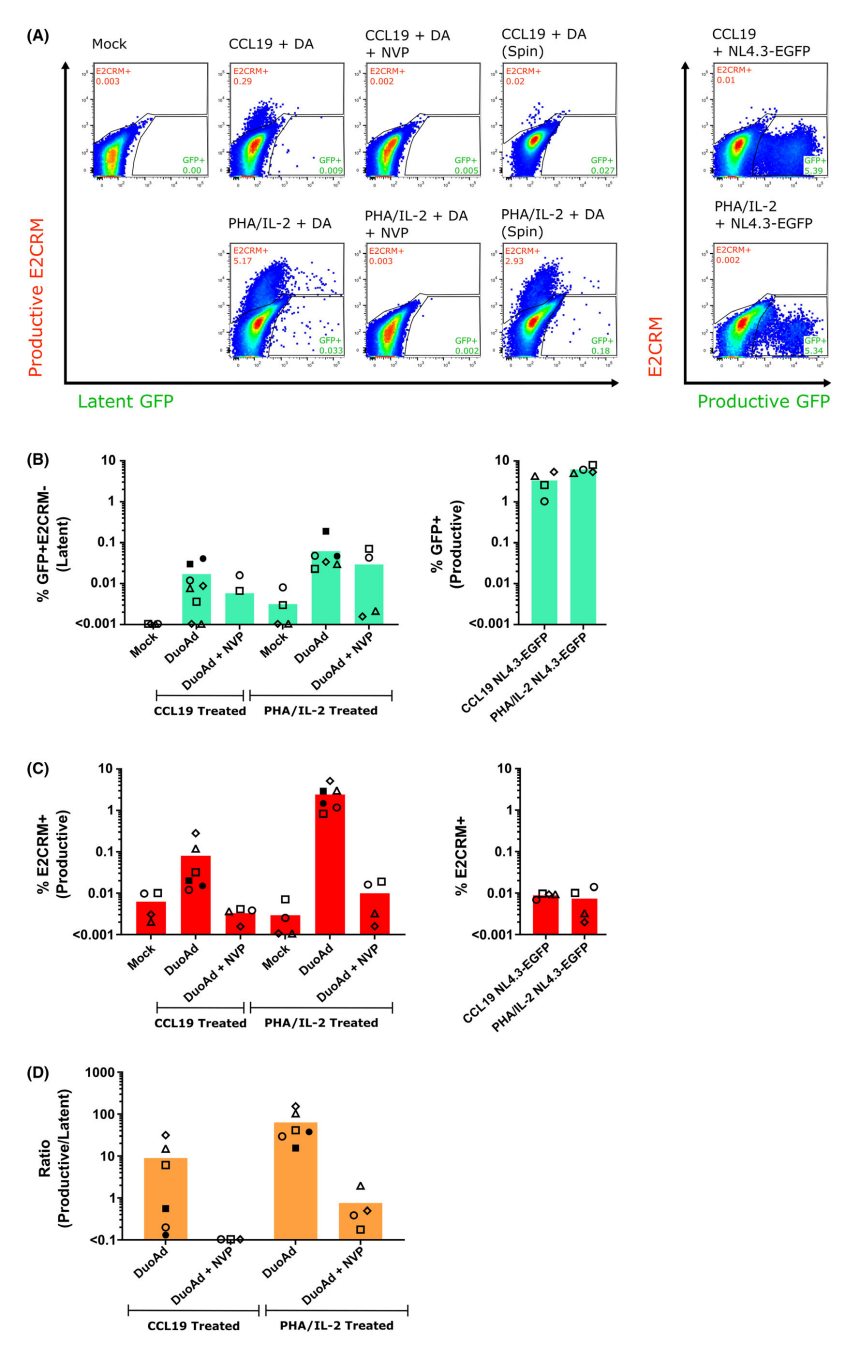
DuoAdvance can infect activated and resting primary CD4+ T cells. DuoAdvance (DuoAd, DA) was added to CCL19‐treated or PHA/IL‐2‐activated primary CD4+ T cells at a TCID_50_ of 0.5 for 2 hours. The cells were washed and then cultured for a further six days. Uninfected cells (mock), cells infected with DuoAd in the presence of Nevirapine (NVP) to block infection, and cells infected with 0.5 TCID_50_ NL4.3‐EGFP as a comparative control were included. In some experiments, spinoculation (spin, closed symbols) was included. (**A**) FACS dot plots from a representative experiment is shown and includes the gating used to determine GFP+E2CRM− (latent) and E2CRM+ (Productive) cells. (**B, C**) Resulting levels of GFP+E2CRM− latent and E2CRM− productive cells from n ≤ 6 experiments are shown. On a separate graph, the levels of GFP+ (productive infection reporter) and E2CRM+ (background) from the NL4.3‐EGFP infection positive control are also shown. (**D**) The ratio of productive to latent infection was calculated using the data from B and C, with the fluorescence background in the mock sample subtracted from the DuoAd‐infected samples before calculating the productive to latent infection ratio. Each symbol represents a different donor and the height of the column represents the mean of the experiments.

We next used DuoAdvance to infect CCL19‐treated and PHA/IL‐2‐activated CD4+ T cells. E2CRM was detected in both models and this declined when nevirapine was added to the cell culture prior to, during and after infection. As expected, the frequency of E2CRM+ cells was higher in PHA/IL‐2 activated (mean (range) = 2.55 (0.83 to 5.14)%) compared to CCL19‐treated cells (mean (range) = 0.11 (0.01 to 0.28)%) (Figure 2A,C and D). Low frequencies of GFP (latent infection) were detected in both CCL19‐treated (mean (range) = 0.01 (0.00 to 0.01)%) and PHA/IL‐2‐activated CD4+ T cells (mean (range) = 0.034 (0.02 to 0.05)%) (Figure 2A‐B). These GFP frequencies were higher than mock infection and when infection was performed in the presence of nevirapine. Spinoculation of the DuoAdvance virus at the time of infection of primary T cells was also tested to enhance viral binding and infection 17; however, we found little change in the number of infected cells following spinoculation (Figure 2B,C, closed symbols) or when we increased the TCID_50_ from 0.5 to 1 (data not shown) to enhance infection.

The low frequency of latently infected cells observed following infection of primary resting CD4+ T cells with DuoAdvance may be explained by inefficient expression of the EF1α‐driven GFP latent reporter in primary resting CD4+ T cells, in contrast to expression in the Jurkat T‐cell line (Figure 1). Promoter silencing may account for the low expression levels in resting CD4+ T cells compared to the actively dividing Jurkat T‐cell line. A key feature of resting CD4+ T cells is sequestration of transcription factors NF‐κB and nuclear factor of activated T cell (NFAT) 18. These transcription factors are important for the initiation of transcription of HIV and may also be important for initiation of transcription of the latent GFP reporter from the EF1α promoter. The downregulation of antiretroviral defence genes such as SAM domain and HD domain‐containing protein 1 (SAMHD1) and guanylate‐binding protein 5 (GBP5) in Jurkat T cells could further contribute to the success of viral infection in cell lines with these virus strains compared to primary CD4+ T cells, where these genes are not downregulated 19. Additionally, the recognition of CG dinucleotides in viral RNA by the cellular zinc‐finger antiviral protein (ZAP) could have contributed to depleted cytoplasmic viral RNA and impaired viral expression in primary resting CD4+ T cells 20.

### 3.3 Inducible virus is detected in E2CRM negative cells

**Figure 3:**
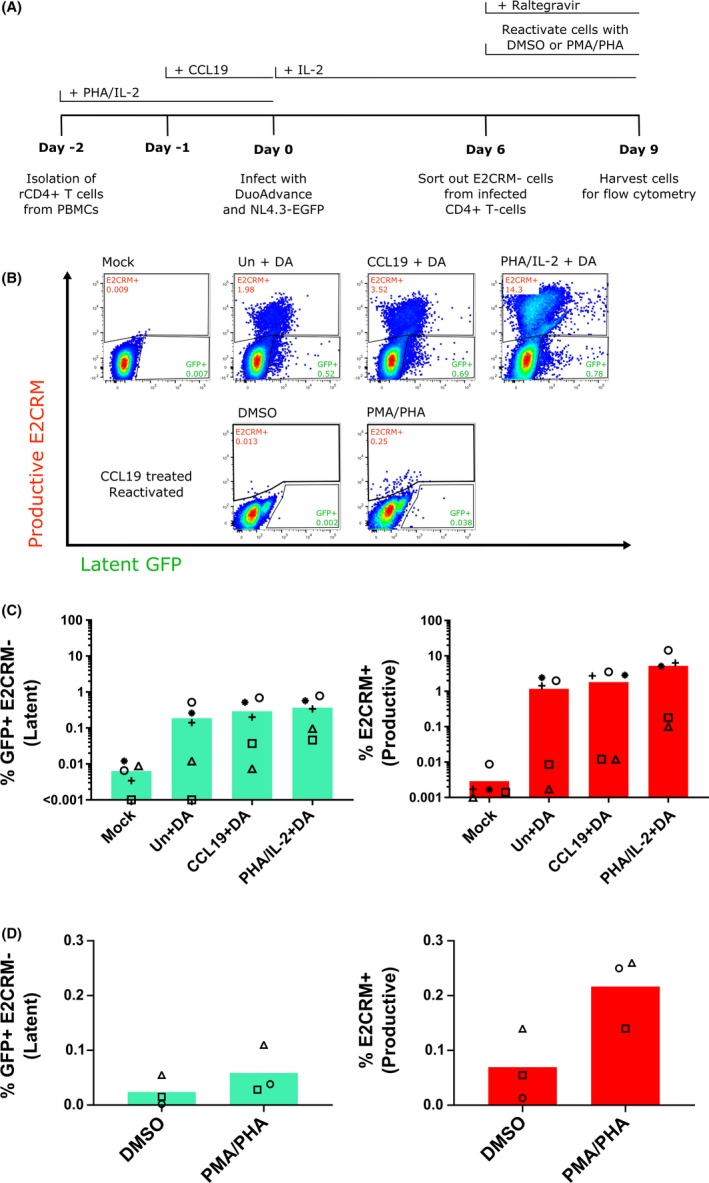
E2 Crimson negative cells harbour inducible latent virus. CD4+ T cells were isolated from PBMC and left resting (unstimulated, Un) or stimulated with either CCL19 or PHA/IL‐2 and then infected with either DuoAdvance (DA) virus or media only (mock) (**A**). After six days, expression of both fluorescent proteins was quantified by flow cytometry (**B‐D**). The E2 Crimson negative cell population (cells that were not present in the E2CRM+ gate in B top panel) was sorted from the CCL19‐treated samples six days after infection and then stimulated with either DMSO or PMA/PHA in the presence of 1 μM raltegravir and expression of both fluorescent proteins quantified by flow cytometry (**D**). Each symbol represents a different donor and the column represents the mean.

We next performed a reactivation experiment (Figure 3A) to test whether the E2CRM cells at six days post‐infection with DuoAdvance contained inducible virus (consistent with latent infection), even if there was poor constitutive expression of GFP. Using cells from different donors, at six days post‐infection with DuoAdvance, low levels of GFP expression were again detected in all conditions (unactivated cells: mean (range) = 0.19 (0.00 to 0.52)%; CCL19 cells: mean (range) = 0.29 (0.01 to 0.69)%; PHA/IL‐2 cells: mean (range) = 0.37 (0.05 to 0.78)%) that was higher than mock infection (mean (range) = 0.01 (0.00 to 0.01)%) (Figure 3B and C *left panel*). There was an increase in E2CRM productive infection in all conditions compared to mock infection (unactivated cells: mean (range) = 1.17 (0.00 to 2.44)%; CCL19 cells: mean (range) = 1.83 (0.01 to 3.52)%; PHA/IL‐2 cells: mean (range) = 5.21 (0.10 to 14.30)%) (Figure 3B and C *right panel*). Infection of PHA/IL‐2 activated cells resulted in the highest levels of latent and productive infection (Figure 3B‐C).

To test for inducible virus, the E2CRM− population was isolated on day 6 from CCL19‐treated infected cells and reactivated with PMA/PHA for three days. PMA/PHA led to an increase in the number of E2CRM+ cells **3.1‐fold** versus the DMSO diluent control (Figure 3D). This revealed that the E2CRM cells sorted six days post‐infection harboured inducible virus, even though these cells had low to absent GFP expression prior to stimulation. Interestingly, the level of GFP+E2CRM− latent cells also increased **2.4‐fold** with PMA/PHA reactivation compared to the DMSO diluent control (Figure 3D), suggesting that the latent reporter may need the cell to be in an activated state to effectively express GFP protein. We do not believe that this increase in GFP was a result of integration of unintegrated DNA following T cell activation as reactivation experiments were performed in the presence of an integrase inhibitor.

In our experiments, the expression of the EF1α‐driven GFP clearly increased following T cell activation. It is worth noting that we were unable to determine if the inefficient expression of GFP in resting T cells was a feature of the EF1α promoter itself or a consequence of the EF1α promoter in the background of the DuoAdvance construct. However, we propose that the use of the EF1α promoter in a dual reporter virus might significantly underestimate the frequency of latently infected cells, particularly when using a pre‐activation model of resting CD4+ T cell latency.

In activated primary CD4+ T cells, low expression of the latent reporter GFP was observed despite efficient expression of the HIV LTR‐driven E2CRM (Figure 2) might be explained by promoter competition. This can occur when a promoter that has a higher affinity for a limiting factor competitively inhibits transcription from an adjacent promoter 21. For example, once the HIV LTR becomes activated by Tat, there may be preferential recruitment of transcription factors to the HIV LTR promoter driving E2CRM expression at the expense of the EF1α promoter and GFP expression, thereby suppressing GFP and resulting in a higher proportion of E2CRM+ cells. Similar promoter competition has been reported in other model systems such as human embryonic stem cells transduced with a dual‐promoter vector 22 and HeLa cells co‐transfected with HIV and HIV Rev binding aptamer under the control of the human cytomegalovirus (CMV) promoter 23.

Collectively, these experiments demonstrate that while DuoAdvance can successfully infect the Jurkat T‐cell lines and establish latent and productive infection, following infection of primary CD4+ T cells, only E2 Crimson expression was detected and there was poor constitutive expression of the EF1α promoter as detected by GFP expression. Expression of E2 Crimson and GFP could be induced following activation of sorted E2CRM‐CCL19 CD4+ T cells suggesting integration did occur in these cells, but GFP had poor constitutive expression and was not expressed in all cells that contained inducible virus.

Interestingly, Chavez and colleagues noted using Duo‐Fluo, that in activated T cells returning to a resting state, the expression of both latent and productive infection fluorescent markers were progressively lost over time 12. The second generation HIV_GKO_ variant of Duo‐Fluo has only been used to infect activated T cells to date and expression of the new EF1α‐driven mKO2 latency reporter has not yet been examined in resting CD4+ T cells 14. Another dual‐fluorescent reporter virus (HIfate‐E) has some similarities to DuoAdvance 24. In this construct the latency reporter was changed to GFP and the productive reporter to E2 Crimson and a composite EF1α‐HTLV‐1 promoter was used in place of the EF1α promoter to drive the GFP latency reporter. Following infection of activated CD4+ T cells with HIfate‐E, insufficient latently infected cells were generated to assess the response to further activation 24, suggesting a very low yield of latently infected cells despite successful infection of activated CD4+ T cells similar to this study. Further investigation with these two new viruses, HIV_GKO_ or HIfate‐E, in direct infection of resting CD4+ T cells will elucidate whether these viruses are suited to studying HIV latency in pre‐activation models.

Developing a sensitive and specific marker of latently infected cells using an in vitro model of a dual reporter virus to infect primary CD4 T cells is of high interest as this would allow for examination of host gene changes following latency using techniques such as scRNAseq as these studies cannot be performed with immortalized cell lines, and activation of the cell to express viral proteins changes the host transcriptome, as recently reported 25. Using DuoAdvance, if latency is defined by GFP expression but not all latently infected cells express GFP, this would underestimate the frequency of latently infected cells. If these cells were being used to screen for LRAs, this would potentially overestimate the efficiency of a particular LRA. In addition, screening would miss latently infected cells that do not react to an LRA.

Adding an internal ribosome entry site (IRES) between the EF1α promoter and the latency reporter might lead to the improved expression of the latency reporter in resting CD4+ T cells by enhancing the reporter gene activity as previously described 26. This approach could be pursued to drive better expression of the latency reporter in resting CD4+ T cells to study HIV latency and reactivation in resting CD4+ T cells 27.

Finally, a limitation of this study was the number of donors analysed. We examined six or fewer donors for each experiment and therefore could not determine whether the data was normally distributed. In preference to using parametric tests, as is often used for small samples, we have only reported the mean fold changes in different experimental conditions and not reported *p* values.

## 4 Conclusions

We generated a modified dual‐fluorescent reporter virus that was able to successfully infect a Jurkat T‐cell line and generate latent and productively infected cell populations. However, DuoAdvance resulted in low levels of expression of the latency reporter in resting CD4+ T cells, using a pre‐activation model of HIV latency. As current dual‐fluorescent reporters are usually studied by infecting activated CD4+ T cells 11, 12, 14, and expression of latent and productive infection reporters can be progressively lost as activated cells return to a resting state 12, dual‐fluorescent reporter viruses may significantly underestimate the frequency of latently infected cells *in vitro*.

## Competing interests

SRL has received investigator‐initiated, industry funded research support from Merck Sciences, Gilead Sciences and Viiv, and provision of reagents from Infinity Pharmaceuticals, Merck Sciences and BMS for investigator‐initiated research. SRL and JA have research collaborations with Merck Sciences unrelated to this work.

## Authors’ contributions

Y.K., S.R.L. and J.L.A. designed the research study. Y.K. and J.L.A. performed the research. Y.K., P.U.C. and J.L.A analysed the data. Y.K., S.R.L and J.L.A wrote and edited the paper. All co‐authors have read and approved the paper.

## Supporting information


**Data S1.** Sequence of DuoAdvance (6668bp) Plasmid Insert.
