# Supplementary material for: Limitations of dual‐fluorescent HIV reporter viruses in a model of pre‐activation latency
Source: J Int AIDS Soc. 2019 Dec 19;22(12):e25425. doi: 10.1002/jia2.25425 (PMC6922067; doi:10.1002/jia2.25425)
Supplement: Supplementary file 1 — Data S1. Sequence of DuoAdvance (6668bp) Plasmid Insert. [file JIA2-22-e25425-s001.docx]

**Sequence of DuoAdvance (6668bp) Plasmid Insert**

AGTAAAACACCATATGTATGTTTCAGGGAAAGCTAGGGGATGGTTTTATAGACATCACTATGAAAGCCCTCATCCAAGAATAAGTTCAGAAGTACACATCCCACTAGGGGATGCTAGATTGGTAATAACAACATATTGGGGTCTGCATACAGGAGAAAGAGACTGGCATTTGGGTCAGGGAGTCTCCATAGAATGGAGGAAAAAGAGATATAGCACACAAGTAGACCCTGAACTAGCAGACCAACTAATTCATCTGTATTACTTTGACTGTTTTTCAGACTCTGCTATAAGAAAGGCCTTATTAGGACACATAGTTAGCCCTAGGTGTGAATATCAAGCAGGACATAACAAGGTAGGATCTCTACAATACTTGGCACTAGCAGCATTAATAACACCAAAAAAGATAAAGCCACCTTTGCCTAGTGTTACGAAACTGACAGAGGATAGATGGAACAAGCCCCAGAAGACCAAGGGCCACAGAGGGAGCCACACAATGAATGGACACTAGAGCTTTTAGAGGAGCTTAAGAATGAAGCTGTTAGACATTTTCCTAGGATTTGGCTCCATGGCTTAGGGCAACATATCTATGAAACTTATGGGGATACTTGGGCAGGAGTGGAAGCCATAATAAGAATTCTGCAACAACTGCTGTTTATCCATTTCAGAATTGGGTGTCGACATAGCAGAATAGGCGTTACTCGACAGAGGAGAGCAAGAAATGGAGCCAGTAGATCCTAGACTAGAGCCCTGGAAGCATCCAGGAAGTCAGCCTAAAACTGCTTGTACCAATTGCTATTGTAAAAAGTGTTGCTTTCATTGCCAAGTTTGTTTCATGACAAAAGCCTTAGGCATCTCCTATGGCAGGAAGAAGCGGAGACAGCGACGAAGAGCTCATCAGAACAGTCAGACTCATCAAGCTTCTCTATCAAAGCAGTAAGTAGTACATGTAATGCAACCTATAATAGTAGCAATAGTAGCATTAGTAGTAGCAATAATAATAGCAATAGTTGTGTGGTCCATAGTAATCATAGAATATAGGAAAATATTAAGACAAAGAAAAATAGACAGGTTAATTGATAGACTAATAGAAAGAGCAGAAGACAGTGGCAATGAGAGTGAAGGAGAAGTATCAGCACTTGTGGAGATGGGGGTGGAAATGGGGCACCATGCTCCTTGGGATATTGATGATCTGTAGTGCTACAGAAAAATTGTGGGTCACAGTCTATTATGGGGTACCTGTGTGGAAGGAAGCAACCACCACTCTATTTTGTGCATCAGATGCTAAAGCCTATGATACAGAGGTACATAATGTTTGGGCCACACATGCCTGTGTACCCACAGACCCCAACCCACAAGAAGTAGTATTGGTAAATGTGACAGAAAATTTTAACATGTGGAAAAATGACATGGTAGAACAGATGCATGAGGATATAATCAGTTTATGGGATCAAAGCCTAAAGCCATGTGTAAAATTAACCCCACTCTGTGTTAGTTTAAAGTGCACTGATTTGAAGAATGATACTAATACCAATAGTAGTAGCGGGAGAATGATAATGGAGAAAGGAGAGATAAAAAACTGCTCTTTCAATATCAGCACAAGCATAAGAGATAAGGTGCAGAAAGAATATGCATTCTTTTATAAACTTGATATAGTACCAATAGATAATGATACTACCAGCTATAGGTTGATAAGTTGTAACACCTCAGTCATTACACAGGCCTGTCCAAAGGTATCCTTTGAGCCAATTCCCATACATTATTGTGCCCCGGCTGGTTTTGCGATTCTAAAATGTAATAATAAGACGTTCAATGGAACAGGACCATGTACAAATGTCAGCACAGTACAATGTACACATGGAATCAGGCCAGTAGTATCAACTCAACTGCTGTTAAATGGCAGTCTAGCAGAAGAAGATGTAGTAATTAGATCTGCCAATTTCACAGACAATGCTAAAACCATAATAGTACAGCTGAACACATCTGTAGAAATTAATTGTACAAGACCCAACAACAATACAAGAAAAAGTATCCGTATCCAGAGGGGACCAGGGAGAGCATTTGTTACAATAGGAAAAATAGGAAATATGAGACAAGCACATTGTAACATTAGTAGAGCAAAATGGAATGCCACTTTAAAACAGATAGCTAGCAAATTAAGAGAACAATTTGGAAATAATAAAACAATAATCTTTAAGCAATCCTCAGGAGGGGACCCAGAAATTGTAACGCACAGTTTTAATTGTGGAGGGGAATTTTTCTACTGTAATTCAACACAACTGTTTAATAGTACTTGGTTTAATAGTACTTGGAGTACTGAAGGGTCAAATAACACTGAAGGAAGTGACACAATCACACTCCCATGCAGAATAAAACAATTTATAAACATGTGGCAGGAAGTAGGAAAAGCAATGTATGCCCCTCCCATCAGTGGACAAATTAGATGTTCATCAAATATTACTGGGCTGCTATTAACAAGAGATGGTGGTAATAACAACAATGGGTCCGAGATCTTCAGACCTGGAGGAGGCGATATGAGGGACAATTGGAGAAGTGAATTATATAAATATAAAGTAGTAAAAATTGAACCATTAGGAGTAGCACCCACCAAGGCAAAGAGAAGAGTGGTGCAGAGAGAAAAAAGAGCAGTGGGAATAGGAGCTTTGTTCCTTGGGTTCTTGGGAGCAGCAGGAAGCACTATGGGCGCAGCGTCAATGACGCTGACGGTACAGGCCAGACAATTATTGTCTGATATAGTGCAGCAGCAGAACAATTTGCTGAGGGCTATTGAGGCGCAACAGCATCTGTTGCAACTCACAGTCTGGGGCATCAAACAGCTCCAGGCAAGAATCCTGGCTGTGGAAAGATACCTAAAGGATCAACAGCTCCTGGGGATTTGGGGTTGCTCTGGAAAACTCATTTGCACCACTGCTGTGCCTTGGAATGCTAGTTGGAGTAATAAATCTCTGGAACAGATTTGGAATAACATGACCTGGATGGAGTGGGACAGAGAAATTAACAATTACACAAGCTTAATACACTCCTTAATTGAAGAATCGCAAAACCAGCAAGAAAAGAATGAACAAGAATTATTGGAATTAGATAAATGGGCAAGTTTGTGGAATTGGTTTAACATAACAAATTGGCTGTGGTATATAAAATTATTCATAATGATAGTAGGAGGCTTGGTAGGTTTAAGAATAGTTTTTGCTGTACTTTCTATAGTGAATAGAGTTAGGCAGGGATATTCACCATTATCGTTTCAGACCCACCTCCCAATCCCGAGGGGACCCGACAGGCCCGAAGGAATAGAAGAAGAAGGTGGAGAGAGAGACAGAGACAGATCCATTCGATTAGTGAACGGATCCTTGGCACTTATCTGGGACGATCTGCGGAGCCTGTGCCTCTTCAGCTACCACCGCTTGAGAGACTTACTCTTGATTGTAACGAGGATTGTGGAACTTCTGGGACGCAGGGGGTGGGAAGCCCTCAAATATTGGTGGAATCTCCTACAATATTGGAGTCAGGAGCTAAAGAATAGTGCTGTTAGCTTGCTCAATGCCACAGCCATAGCAGTAGCTGAGGGGACAGATAGGGTTATAGAAGTAGTACAAGGAGCTTGTAGAGCTATTCGCCACATACCTAGAAGAATAAGACAGGGCTTGGAAAGGATTTTGCTATAAGCATGGATAGCACTGAGAACGTCATCAAGCCCTTCATGCGCTTCAAGGTGCACATGGAGGGCTCCGTGAACGGCCACGAGTTCGAGATCGAGGGCGTGGGCGAGGGCAAGCCCTACGAGGGCACCCAGACCGCCAAGCTGCAAGTGACCAAGGGCGGCCCCCTGCCCTTCGCCTGGGACATCCTGTCCCCCCAGTTCTTCTACGGCTCCAAGGCGTACATCAAGCACCCCGCCGACATCCCCGACTACCTCAAGCAGTCCTTCCCCGAGGGCTTCAAGTGGGAGCGCGTGATGAACTTCGAGGACGGCGGCGTGGTGACCGTGACCCAGGACTCCTCCCTGCAGGACGGCACCCTCATCTACCACGTGAAGTTCATCGGCGTGAACTTCCCCTCCGACGGCCCCGTAATGCAGAAGAAGACTCTGGGCTGGGAGCCCTCCACTGAGCGCAACTACCCCCGCGACGGCGTGCTGAAGGGCGAGAACCACATGGCGCTGAAGCTGAAGGGCGGCGGCCACTACCTGTGTGAGTTCAAGTCCATCTACATGGCCAAGAAGCCCGTGAAGCTGCCCGGCTACCACTACGTGGACTACAAGCTCGACATCACCTCCCACAACGAGGACTACACCGTGGTGGAGCAGTACGAGCGCGCCGAGGCCCGCCACCACCTGTTCCAGTAGAGCGACCCTCGAGTACTAGGATCCATTAGGCGGCCGCGGATCTGCGATCGCTCCGGTGCCCGTCAGTGGGCAGAGCGCACATCGCCCACAGTCCCCGAGAAGTTGGGGGGAGGGGTCGGCAATTGAACCGGTGCCTAGAGAAGGTGGCGCGGGGTAAACTGGGAAAGTGATGTCGTGTACTGGCTCCGCCTTTTTCCCGAGGGTGGGGGAGAACCGTATATAAGTGCAGTAGTCGCCGTGAACGTTCTTTTTCGCAACGGGTTTGCCGCCAGAACACAGCTGAAGCTTCGAGGGGCTCGCATCTCTCCTTCACGCGCCCGCCGCCCTACCTGAGGCCGCCATCCACGCCGGTTGAGTCGCGTTCTGCCGCCTCCCGCCTGTGGTGCCTCCTGAACTGCGTCCGCCGTCTAGGTAAGTTTAAAGCTCAGGTCGAGACCGGGCCTTTGTCCGGCGCTCCCTTGGAGCCTACCTAGACTCAGCCGGCTCTCCACGCTTTGCCTGACCCTGCTTGCTCAACTCTACGTCTTTGTTTCGTTTTCTGTTCTGCGCCGTTACAGATCCAAGCTGTGACCGGCGCCTACGCTAGCGCTACCGGTCGCCACCATGGTGAGCAAGGGCGAGGAGCTGTTCACCGGGGTGGTGCCCATCCTGGTCGAGCTGGACGGCGACGTAAACGGCCACAAGTTCAGCGTGTCCGGCGAGGGCGAGGGCGATGCCACCTACGGCAAGCTGACCCTGAAGTTCATCTGCACCACCGGCAAGCTGCCCGTGCCCTGGCCCACCCTCGTGACCACCCTGACCTACGGCGTGCAGTGCTTCAGCCGCTACCCCGACCACATGAAGCAGCACGACTTCTTCAAGTCCGCCATGCCCGAAGGCTACGTCCAGGAGCGCACCATCTTCTTCAAGGACGACGGCAACTACAAGACCCGCGCCGAGGTGAAGTTCGAGGGCGACACCCTGGTGAACCGCATCGAGCTGAAGGGCATCGACTTCAAGGAGGACGGCAACATCCTGGGGCACAAGCTGGAGTACAACTACAACAGCCACAACGTCTATATCATGGCCGACAAGCAGAAGAACGGCATCAAGGTGAACTTCAAGATCCGCCACAACATCGAGGACGGCAGCGTGCAGCTCGCCGACCACTACCAGCAGAACACCCCCATCGGCGACGGCCCCGTGCTGCTGCCCGACAACCACTACCTGAGCACCCAGTCCGCCCTGAGCAAAGACCCCAACGAGAAGCGCGATCACATGGTCCTGCTGGAGTTCGTGACCGCCGCCGGGATCACTCTCGGCATGGACGAGCTGTACAAGTAACTCATGAGCTGTAGATCTTAGCCACTTTTTAAAAGAAAAGGGGGGACTGGAAGGGCTAATTCACTCCCAACGAAGACAAGATATCCTTGATCTGTGGATCTACCACACACAAGGCTACTTCCCTGATTGGCAGAACTACACACCAGGGCCAGGGATCAGATATCCACTGACCTTTGGATGGTGCTACAAGCTAGTACCAGTTGAGCAAGAGAAGGTAGAAGAAGCCAATGAAGGAGAGAACACCCGCTTGTTACACCCTGTGAGCCTGCATGGGATGGATGACCCGGAGAGAGAAGTATTAGAGTGGAGGTTTGACAGCCGCCTAGCATTTCATCACATGGCCCGAGAGCTGCATCCGGAGTACTTCAAGAACTGCTGACATCGAGCTTGCTACAAGGGACTTTCCGCTGGGGACTTTCCAGGGAGGCGTGGCCTGGCGGGACTGGGGAGTGGCGAGCCCTCAGATGCTGCATATAAGCAGCTGCTTTTTGCTTGTACTGGGTCTCTCTGGTTAGACCAGATCTGAGCCTGGGAGCTCTCTGGCTAACTAGGGAACCCACTGCTTAAGCCTCAATAAAGCTTGCCTTGAGtgcttcaagtagtgtgtgcccgtctgttgtgtgactctggtaactagagatccctcagacccttttagtcagtgtggaaaatctctagcaGTAGTAGTTCATGTCATCTTATTATTCAGTATTTATAACTTGCAAAGAAATGAATATCAGAGAGTGAGAGGCCTTGACATTATAATAGATTTAGCAGGAATTGAACTAGGAGTGGAGCACACAGGCAAAGCTGCAGAAGTACTTGGAAGAAGCCACCAGAGATACTCACGATTCTGCACATACCTGGCTAATCCCAGATCCTAAGGATTACATTAAGTTTACTAACATTTATATAATGATTTATAGTTTAAAGTATAAACTTATCTAATTTACTATTCTGACAGATATTAATTAATCCTCAAATA

Red- Nucleotide sequence of E2 Crimson

Yellow- Nucleotide sequence of EF1α promoter

Green- Nucleotide sequence of EGFP
